# Neoadjuvant everolimus plus letrozole versus fluorouracil, epirubicin and cyclophosphamide for ER-positive, HER2-negative breast cancer: study protocol for a randomized pilot trial

**DOI:** 10.1186/s13063-017-2228-5

**Published:** 2017-10-25

**Authors:** Wei Wu, Heran Deng, Nanyan Rao, Na You, Yaping Yang, Minghui Cao, Jieqiong Liu

**Affiliations:** 1 Guangdong Provincial Key Laboratory of Malignant Tumor Epigenetics and Gene Regulation, Breast Tumor Center, Sun Yat-Sen Memorial Hospital, Sun Yat-Sen University, Yanjiang West Road 107#, Guangzhou, 510120 China; 2 Department of Statistical Science, School of Mathematics and Southern China Research Center of Statistical Science, Sun Yat-Sen University, Guangzhou, 510275 China; 3 Guangdong Provincial Key Laboratory of Malignant Tumor Epigenetics and Gene Regulation, Department of Anesthesiology, Sun Yat-Sen Memorial Hospital, Sun Yat-Sen University, Yanjiang West Road 107#, Guangzhou, 510120 China

**Keywords:** Randomized neoadjuvant pilot trial, Everolimus plus letrozole, Fluorouracil epirubicin and cyclophosphamide (FEC), ER-positive, HER2-negative breast cancer

## Abstract

**Background:**

The response to neoadjuvant chemotherapy (NAC) varies by estrogen receptor (ER) and human epidermal growth factor receptor 2 (HER2) statuses, with responses being lower in ER-positive, HER2-negative tumors as compared with ER-negative, HER2-positive or triple-negative tumors. Neoadjuvant endocrine therapy (NET) is an attractive alternative to NAC for ER-positive, HER2-negative cancer. However, a prior trial comparing NET with standard NAC in ER-positive tumor showed that the difference of response was not significant. Studies demonstrated that the mTOR inhibitor everolimus could sensitize breast tumors to endocrine therapy. A pilot open-label, randomized trial has been designed to evaluate the feasibility, efficacy and tolerability of neoadjuvant everolimus plus letrozole versus NAC in treating postmenopausal women with ER-positive, HER2-negative breast cancer.

**Methods:**

Forty postmenopausal women with non-metastatic ER-positive, HER2-negative invasive breast cancer with a primary tumor > 2 cm or positive axillary lymph node(s) proved by biopsy will be randomly (1:1) enrolled from Sun Yat-Sen Memorial Hospital to receive neoadjuvant everolimus plus letrozole for 18 weeks or fluorouracil, epirubicin plus cyclophosphamide (FEC) for six cycles before surgery. Primary outcome is the feasibility of the trial. Secondary outcome measures include ultrasound response rate, pathological complete response rate, breast-conserving surgery rate, toxicities, and changes in the percentages of peripheral blood CD4^+^ T cells, CD8^+^ T cells, T helper cells, regulatory T cells, and NK cells.

**Discussion:**

This is the first study to determine the feasibility, efficacy and tolerability of head-to-head neoadjuvant everolimus plus letrozole versus neoadjuvant FEC in treating postmenopausal women with ER-positive, HER2-negative breast cancer. The trial will provide evidence to assess the feasibility of a future multicenter, randomized controlled trial, and will provide valuable clinical data of the immunoregulatory effect of everolimus in breast cancer.

**Trial registration:**

ClinicalTrials.gov registry, ID: NCT02742051. Registered on 7 April 2016.

**Electronic supplementary material:**

The online version of this article (doi:10.1186/s13063-017-2228-5) contains supplementary material, which is available to authorized users.

## Background

Neoadjuvant chemotherapy (NAC) is considered as the standard of treatment in the management of locally advanced breast cancer, and one of the main effects of NAC is its potential to downstage the pathological extent of disease, which makes lumpectomy plus radiotherapy become an alternative option to mastectomy [1]. Nevertheless, the response to NAC varies by estrogen receptor (ER) and human epidermal growth factor receptor 2 (HER2) statuses, with responses being lower in ER-positive, HER2-negative tumors as compared with ER-negative, HER2-positive or triple-negative tumors as evidenced by significantly lower pathological complete response (pCR) rates [2, 3]. ER-positive, HER2-negative breast cancer accounts for approximately 72.7% of all breast cancer cases based on the National Cancer Institute’s Surveillance, Epidemiology and End Results (SEER) data [4], but its pCR rate after NAC is less than 15% [2]. Neoadjuvant endocrine therapy (NET) is an attractive alternative to NAC for ER-positive, HER2-negative tumors, and randomized trials demonstrated that NET with the third-generation aromatase inhibitors (AIs) was superior to tamoxifen in terms of overall response rates and operability for postmenopausal women [5, 6]. However, a multicenter, randomized phase 2 trial comparing NET (the third-generation AI letrozole) with standard NAC (fluorouracil, epirubicin plus cyclophosphamide, FEC) in ER-positive postmenopausal breast cancer showed that the radiological objective response was 59.1% (95% CI 36.4–79.3%) with letrozole, which was a little bit higher than that (54.5% (95% CI 32.2–75.6%)) with FEC chemotherapy, but the difference was not statistically significant [7]. Therefore, in order to increase the response rate of ER-positive, HER2-negative breast cancer to NET, it is critical to find other targeted drugs combined with AIs that could sensitize breast tumors to endocrine therapy.

The PI3K/Akt/mTOR pathway modulates responses to signals communicated through the ER and HER family of receptors in breast cancer, and this pathway is critical in the clinical sensitivity of breast tumor to endocrine therapy [8–11]. For postmenopausal patients with ER-positive advanced breast cancer, the orally administered mTOR inhibitor everolimus combined with an AI was recently shown to prolong progression-free survival (PFS) relative to placebo combined with an AI from 2.8 months to 6.9 months (*p* < 0.001) in the phase 3 BOLERO-2 trial [12]. In the neoadjuvant setting, a phase 2, randomized, placebo-controlled trial showed that the clinical response rate with 4 months neoadjuvant everolimus plus letrozole was significantly higher than that with letrozole plus placebo (68.1 vs. 59.1%) [13]. In addition, the safety profile was consistent with historical results of everolimus monotherapy in this trial [13]. Thus, we hypothesized that for postmenopausal patients with ER-positive, HER2-negative breast cancer, neoadjuvant everolimus plus letrozole could improve clinical response rate, pCR rate, and breast-conserving rate compared with neoadjuvant FEC chemotherapy. In addition, the mTOR pathway also plays an important role in immunoregulation. Inhibition of mTOR has been shown to result in expansion of immunosuppressive regulatory T cells (Tregs) in patients with metastatic renal cell carcinoma or prostate cancer [14–17]. In breast cancer, there is no clinical studies focusing on the immunoregulatory effects of mTOR inhibitors so far; only a preclinical study found that everolimus decreased the number of CD8^+^ T cells but had no effect on CD4^+^ T and natural killer (NK) cells in four T1 tumor-bearing mice compared to the control group [18].

Based on these data, the aim of the present open-label, randomized feasibility trial is to evaluate the feasibility, efficacy and tolerability of neoadjuvant everolimus plus letrozole versus neoadjuvant FEC chemotherapy in treating postmenopausal women with ER-positive, HER2-negative breast cancer. Due to the envisaged recruitment challenges and uncertain intervention acceptability, prior to undertaking large comparative trials, this trial was designed with an integrated pilot phase. Effects of everolimus plus letrozole on the percentages of peripheral blood CD4^+^ T cells, CD8^+^ T cells, T helper cells (Ths), Tregs, and NK cells will also be assessed.

## Methods

### Study design

**Fig. 1:**
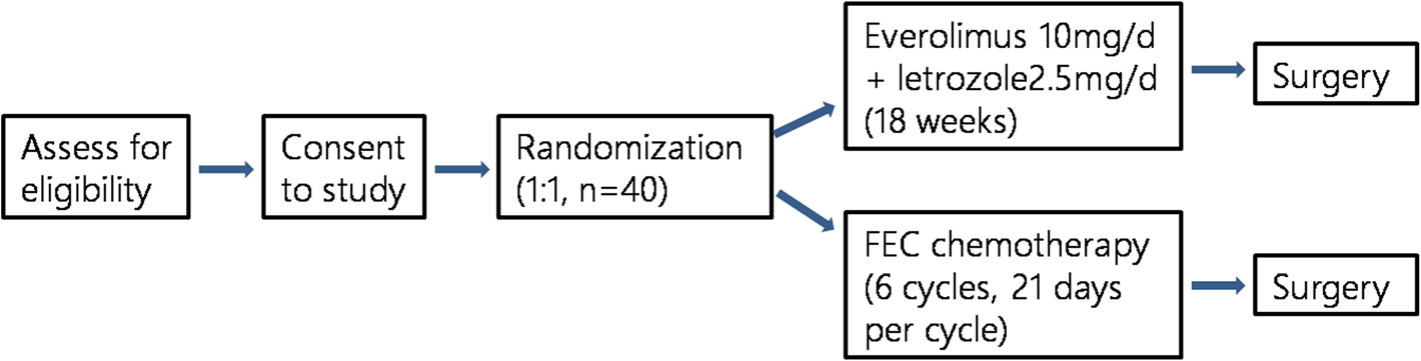
Study design flowchart

**Fig. 2:**
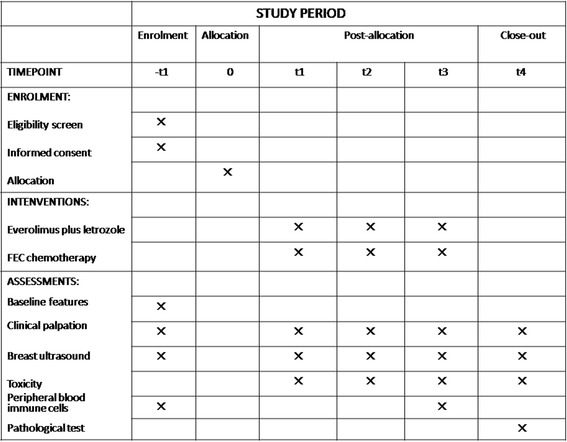
Schedule of enrollment, interventions, and assessments (SPIRIT checklist)

This phase II trial is a single-center, open, randomized pilot feasibility study. Forty postmenopausal stage M0, ER-positive, HER2-negative invasive breast cancer women who have a primary tumor > 2 cm by imaging or positive axillary lymph node(s) proved by biopsy will be randomly (1:1) enrolled to receive neoadjuvant everolimus plus letrozole for 18 weeks or neoadjuvant FEC for six cycles (every 21 days per cycle) before definitive surgery (study design flowchart is shown in Fig. 1). The schedule of trial enrollment, interventions, and assessments is presented in Fig. 2 (SPIRIT checklist). This protocol was written following the Standard Protocol Items: Recommendations for Interventional trials (SPIRIT) checklist (see Additional file 1).

### Setting

The study is initiated by the Breast Tumor Center of the Sun -Sen Memorial Hospital, and will be conducted in this hospital. Recruitment to the trial has been proceeding for two and a half years.

### Primary objectives

The primary objective is to determine the feasibility of a larger comparative trial, established by composite analysis of elements of eligibility, recruitment, attrition, protocol adherence, missing data, and effect size estimates to allow sample size calculation for the following larger randomized controlled trial.

### Secondary objectives

Secondary objectives are to compare the ultrasound response rate, pCR rate, breast-conserving surgery rate, toxicities, and changes in the percentages of peripheral blood CD4^+^ T cells, CD8^+^ T cells, Ths, Tregs, and NK cells (pre- versus post- neoadjuvant therapy).

### Eligibility criteria

1. The patients signed the written informed consent.
2. The patients present with non-metastatic unilateral invasive ER-positive, HER2-negative breast cancer with a primary breast tumor > 2 cm by imaging or positive axillary lymph node(s) proved by biopsy.
3. Postmenopausal women less than 70 years old.
4. The patients have no history of hormone therapy, chemotherapy, immunotherapy, breast cancer surgery, and radiotherapy.
5. The patients have normal cardiac functions by echocardiography.
6. Eastern Cooperative Oncology Group (ECOG) scores 0–2.
7. The patients are able to receive oral medication.
8. Adequate hematologic function: hemoglobin ≥ 90 g/L; white blood cell (WBC) ≥ 4.0 × 10^9^/L; neutrophils ≥ 1.5 × 10^9^/L; platelets ≥ 100 × 10^9^/L.
9. Adequate hepatic function: alanine transaminase (ALT) and aspartate transaminase (AST) ≤ triple of normal upper limit; total bilirubin (TBIL) ≤ 1.5 times of normal upper limit.
10. Adequate renal function: creatinine ≤ 1.5 times of normal upper limit.

### Exclusion criteria

1. The patients have other cancers at the same time or have the history of other cancers except controlled skin basal cell carcinoma, skin squamous cell carcinoma, or carcinoma in situ of cervix uterus.
2. The patients have active infections that were not suitable for chemotherapy.
3. The patients have severe noncancerous diseases.
4. The patients have bilateral breast cancers or multifocal breast cancers or inflammatory breast cancers.
5. The patients have a history of prior treatment with mTOR inhibitors.
6. The patients are undergoing current administration of anticancer therapies, or are attending other clinical trials.
7. The patients are in some special conditions that they cannot understand the written informed consent, such as they are demented or uncooperative.
8. The patients have allergic history or contraindication of any of the interventional drugs.

### Ethics

The Sun Yat-Sen Memorial Hospital Institutional Review Board approved the study (Protocol number 201608), which was conducted in accordance with the principles of Good Clinical Practice, and the provisions of the Declaration of Helsinki. Written informed consent is obtained from all patients prior to trial participation.

### Safety

All adverse events encountered during the study will be reported on case report form (CRF) and RedCap online database. Adverse events were graded according to National Cancer Institute Common Toxicity Criteria (version 4.0). In case of a serious adverse event (SAE), the physician and research assistant have a legal requirement to inform the central data management office within 24 hours. All SAEs must be reported on the Adverse Event page of the CRF and RedCap online database as well. The principal investigator is responsible for the management of the safety reporting according to local guidelines. The study drug license holder will be provided with copies of all report submissions to regulatory authorities and to the ethics committee that has approved the study. All reported adverse events will be discussed by an independent data monitoring committee.

### Monitoring

The study progress, safety data, and data quality will be monitored by an Independent Data (and safety) Monitoring Board (IDMB), which will be independent of the trial organizer. Safety analysis will be performed on a regular basis and the IDMB will report their findings to the principal investigator. Once every 6 months throughout the trial, the principal investigator will submit a safety report to the accredited Medical Research Ethics Committee (METC), including a list of all suspected SAEs, and an aggregated summary table of all reported SAEs.

### Sample size calculation

Because of the exploratory (pilot) nature of this study, statistical power was not calculated to assess specific study outcomes. The primary aim of this trial is to determine the feasibility of a larger comparative trial. In this pilot trial, 40 patients (20 per group) will be recruited. This sample size calculation was developed in accordance with the recommendations by previous reports on sample size determination for pilot studies [19–21]. This pilot feasibility trial will then provide evidence to inform sample size calculation for a potential subsequent larger randomized controlled trial.

### Statistical analysis

Statistical analysis in this trial will be performed according to the intention-to-treat (ITT) principle. All patients in this study that have taken their study medication on at least one occasion are considered evaluable patients. The ultrasound response rate, pCR rate, breast-conserving surgery rate, and toxicities will be compared between the two arms using the chi-square test. Statistical analyses will be conducted using STATA 12.0 software (StataCorp, College Station, TX, USA). All statistical tests will be two-sided, and statistical significance is defined as *p* < 0.05.

### Randomization

Participants are randomly assigned to the neoadjuvant everolimus plus letrozole and neoadjuvant FEC arm with a 1:1 allocation ratio. Random assignment was done with a computer-assisted randomization-allocation sequence with a block size of four.

### Follow-up

Baseline evaluations are to be conducted within 2 weeks prior to the start of protocol therapy, and comprise medical history, physical examination, toxicity assessment, hematology, serum chemistry, immune monitoring, electrocardiogram, breast ultrasound, mammography, tumor biopsy, and urine human chorionic gonadotrophin.

Patient visits will be scheduled at 6, 12 and 18 weeks, and the preoperative time point. At each visit medical history, physical examination, breast ultrasound, toxicity assessment, hematology, and serum chemistry will be performed.

Clinical palpation and breast ultrasound will be required at fixed time points: baseline, 6, 12 and 18 weeks, and preoperative.

Immune monitoring on peripheral blood CD4^+^ T cells, CD8^+^ T cells, Ths, Tregs, and NK cells will be performed at baseline and subsequently at 18 weeks after the start of the study treatment period (after the completion of neoadjuvant therapy).

Adverse events will be reported during the study treatment phase. Medically significant adverse events considered related to the interventional drugs will be followed up until resolved or considered stable.

### Treatment program and dose modification

Forty postmenopausal women with stage M0, ER-positive, HER2-negative invasive breast cancer who have a primary tumor > 2 cm by imaging or positive axillary lymph node(s) proved by biopsy will be randomly (1:1) enrolled to receive neoadjuvant everolimus (10 mg/d, orally (po)) plus letrozole (2.5 mg/d, po) for 18 weeks or neoadjuvant FEC (fluorouracil 600 mg/m^2^, intravenously (iv), d1 + epirubicin 90 mg/m^2^, iv, d1 + cyclophosphamide 600 mg/m^2^, iv, d1; every 21 days per cycle) for 6 cycles before definitive surgery. In addition, prophylactic use of dexamethasone mouth wash can be used to reduce the everolimus-induced stomatitis in patients who receive neoadjuvant everolimus plus letrozole based on the result of the SWISH trial [22].

Treatment with everolimus will be interrupted for grade 1 thrombocytopenia that lasted longer than 2 weeks and for ≥ grade 2 stomatitis or pneumonitis, any grade 3 hematologic or other nonhematologic toxicity. Treatment will resume with reduced dose of everolimus (5 mg/day) if recovery (defined as grade 1, or normal levels for platelets) occurred. If toxicity persists for more than 4 weeks or reappear after resolution, or if any grade 4 toxicity occurs, treatment with everolimus will be discontinued, and the patients will continue on letrozole until surgery. The maximum permitted dose delay or interruption during chemotherapy is 4 weeks to allow recovery from severe toxicity or for unscheduled procedures. If neutropenic fever or sepsis occurs after a cycle of chemotherapy, the next cycle will be delayed until the absolute neutrophil count is at least 1.0 × 10^9^ cells per liter. After a delay, either dose reduction of all drugs to 80%, or granulocyte colony-stimulating factor (GCSF) support with 100% dose are allowed, and all remaining cycles of the same three-cycle block will be given at those doses. For persistent thrombocytopenia, the next cycle will be delayed until platelets have recovered to at least 100 × 10^9^ cells per liter, and chemotherapy doses will be reduced to 80%, maintaining this dose reduction for subsequent cycles.

### Dissemination

Reports will follow the international guideline: the CONSORT statement. Research findings will be submitted for publication in peer-reviewed journals regardless of whether or not they are statistically significant. Authors will be individuals who have made key contributions to study design and conduct. Trial findings will be submitted for presentation at international breast cancer conferences.

## Discussion

To the best of our knowledge, this is the first study to determine the feasibility, efficacy and tolerability of head-to-head neoadjuvant everolimus plus letrozole versus neoadjuvant FEC in treating postmenopausal women with ER-positive, HER2-negative breast cancer. The study will provide evidence to assess the relevance and feasibility of a larger future multicenter, randomized controlled trial. In addition, mTOR inhibitors such as everolimus have been shown to result in expansion of Tregs in patients with metastatic renal cell carcinoma or prostate cancer [14–17]. While in breast cancer, there are no clinical studies assessing the immunoregulatory effects of mTOR inhibitors. Therefore, this trial will be the first study to provide valuable clinical data of the impact of everolimus on several important peripheral blood immune cells, including CD4^+^ T cells, CD8^+^ T cells, Ths, Tregs, and NK cells in patients with breast cancer.

## Trial status

This trial opened for recruitment June 2016, with recruitment expected to be completed by January 2019. At the time of submission of this study protocol, 50 potentially eligible patients have been approached, 22 were found to be ineligible, 10 patients were enrolled and 6 patient have completed the study. This protocol version number is version 6.0.

## Additional file

Additional file 1: SPIRIT Checklist. Checklist of the Standard Protocol Items: Recommendations for Interventional Trials (SPIRIT) guidelines. (DOCX 49 kb)

## Abbreviations

AIs: Aromatase inhibitors
ALT: Alanine transaminase
AST: Aspartate transaminase
CRF: Case report form
ECOG: Eastern Cooperative Oncology Group
ER: Estrogen receptor
FEC: Fluorouracil, epirubicin plus cyclophosphamide
GCSF: Granulocyte colony-stimulating factor
HER2: Human epidermal growth factor receptor 2
IDMB: Independent Data (and safety) Monitoring Board
ITT: Intention-to-treat
NAC: Neoadjuvant chemotherapy
NET: Neoadjuvant endocrine therapy
NK: Natural killer
pCR: Pathological complete response
SAE: Serious adverse event
SEER: Surveillance, Epidemiology and End Results
TBIL: Total bilirubin
Ths: T helper cells
Tregs: Regulatory T cells
WBC: White blood cell
